# Yeast quality in juvenile diet affects *Drosophila melanogaster* adult life traits

**DOI:** 10.1038/s41598-018-31561-9

**Published:** 2018-08-30

**Authors:** Cédric Grangeteau, Fairouz Yahou, Claude Everaerts, Sébastien Dupont, Jean-Pierre Farine, Laurent Beney, Jean-François Ferveur

**Affiliations:** 1 University Bourgogne Franche-Comté, AgroSup Dijon, PAM UMR A 02.102, F-21000 Dijon, France; 2 Université de Bourgogne Franche-Comté, Centre des Sciences du Goût et de l’Alimentation, AgroSup-UMR 6265 CNRS, UMR 1324 INRA, 6, Bd Gabriel, F-21000 Dijon, France

## Abstract

Diet quality is critical for animal development and survival. Fungi can provide nutrients that are essential to organisms that are unable to synthetize them, such as ergosterol in *Drosophila melanogaster*. Drosophila studies examining the influence of yeast quality in the diet have generally either provided the diet over the whole life span (larva to adult) or during the adult stage and have rarely focussed on the juvenile diet. Here, we tested the effect of yeast quality in the larval diet on pre-adult development and adult weight, survival, reproduction and food preference. The yeast *Saccharomyces cerevisiae* was added in three forms in three treatments—live, heated or dried—to food used as the juvenile diet or was not added (empty treatment). Adults resulting from the larvae raised on these four juvenile diets were all maintained on a similar standard laboratory food diet. Our data indicate that yeast quality in the juvenile diet affects larva-to-pupa—but not pupa-to-adult—development. Importantly, adult survival, food preference, mating behaviour and cuticular pheromones strongly varied with the juvenile diet. Therefore, the variation of yeast quality in the pre-adult Drosophila diet affects key adult life traits involved in food search, reproduction and survival.

## Introduction

Animal survival and fitness depends on the quantity and quality of the diet. Food must be consumed in sufficient amounts and contain essential nutrients to avoid cellular dysfunction and exposure to pathologies^1,2^. Unlike vertebrates, insects are auxotrophic for sterols, which requires them to obtain them from their diet. In particular, insects need to ingest yeasts and yeast-like fungi at both the larval and adult stages to correctly develop^3^. The ingestion of the yeast *Saccharomyces cerevisiae* throughout life was shown to affect various life traits in *Drosophila melanogaster*: a decreased amount of yeast in the larval diet delayed pupal eclosion and produced smaller adults^4^. Similarly, yeast restriction in the adult diet has been shown to decrease resistance to external stresses (cold temperature, starvation, infection)^5,6^. Moreover, a poor-yeast diet throughout life was found to decrease fertility^7,8^ and increase life span^5,9^; these two effects are linked^10^. Many *D*. *melanogaster* studies have shown that the quality of the fungal diet, provided either throughout life or only at the adult age, can affect adult life traits. For example, adults fed with frozen or heat-killed yeast showed altered development, lower fertility, shorter life span and increased sensitivity to stress than did adults fed on a fresh yeast diet^11^. Furthermore these effects persisted even when a 600-fold increased amount of heat-killed yeast was added to the diet^12^. A strong yeast restriction in the larval diet was also shown to increase adult life span^13^.

Larvae and adults of different *Drosophila* species (*D*. *affinis*, *D*. *miranda*, *D*. *persimilis and D*. *pseudoobscura*) are attracted to food sources containing yeast, and each species shows a marked preference for different yeast species^14^. While larval food preference can vary according to their earlier experience and the presence of maternally transmitted microbes^15,16^, the relationship between yeast in the larval diet and adult life traits has rarely been investigated^13^. Here, we measured the influence of pre-adult fungal diet quality on various adult life traits. We discovered that *D*. *melanogaster* larvae fed on different diets containing or lacking *S*. *cerevisiae* yeast in different forms showed differences in pre-adult development and adult life traits including survival, weight, reproduction, cuticular pheromones and food preference.

## Results

We evaluated the effects of yeast quality in the juvenile diet both on pre-imaginal development and on several *D*. *melanogaster* adult traits (aging, weight, food preference, reproductive behaviour and cuticular pheromones). Larvae were raised on three diets containing *S*. *cerevisiae* yeast subjected to different treatments (live wild-type = WT; industrially desiccated = Ind; heated at 90° = 90°) or on a yeast-free diet (øY). After adult emergence, all of the flies were maintained on “Ind” food. Fly groups were named according to their juvenile diet (e.g., “WT” flies were reared on WT-yeast food as larvae).

### Preimaginal development

**Figure 1:**
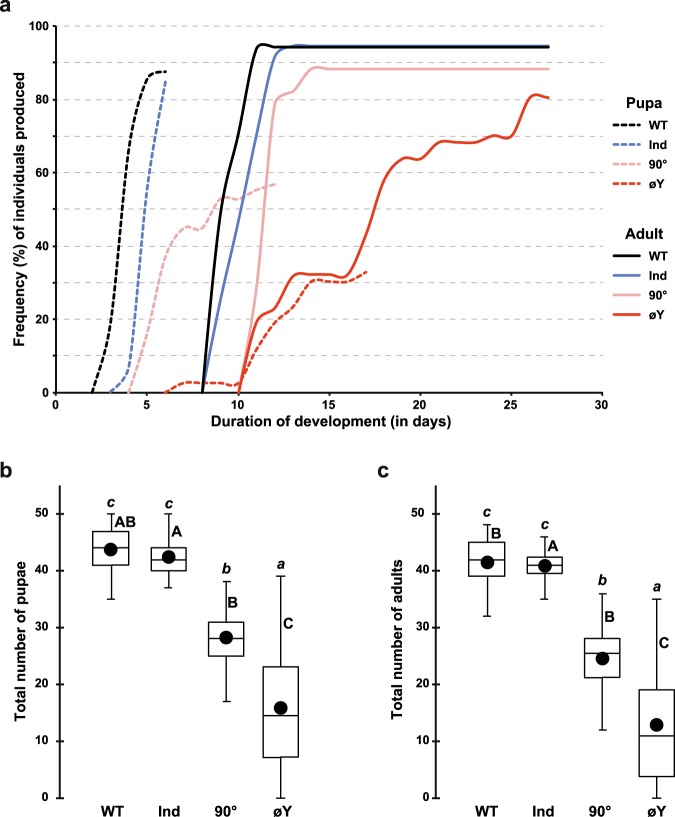
Preimaginal development of *Drosophila melanogaster* individuals fed on juvenile diets containing different preparations of *Saccharomyces cerevisiae* yeast. (**a**) Duration of development (in days) from egg to pupa (dashed lines) or egg to adult (plain lines) and frequency (%) of individuals reared on juvenile diets involving one of four alternative yeast treatments, which are indicated by different colours: live wild-type (WT; black), industrially dried (Ind; blue), 90° heating (90°) and no yeast (øY). Note that adult frequency was calculated based on pupae number. (**b**) Total number of pupae (**c**) and adults resulting from 50 eggs that developed on each of the four juvenile diets. Box-plots represent the 50% median data (the small horizontal bar indicates the median value, and the solid dot represents the mean). The whiskers shown below and above each box represent the first and third quartiles, respectively. Different italic lowercase letters above whiskers indicate significant differences between means (Kruskal Wallis test K(3 df) = 141.1, *p* < 10^−4^, and K(3 df) = 140.1, *p* < 10^−4^). Different uppercase letters next to whiskers indicate significant differences between variances (Levene’s test F(3,175) = 29.4, *p* < 10^−4^ and F(3,175) = 23.6, *p* < 10^−4^). *N* = 48, 39, 42 and 50.

Each food vial was initially seeded with 50 fertilized eggs. For each treatment and vial, we recorded the number of eggs developing into pupae and the duration of this developmental process. Eggs raised on WT and Ind diets produced 88 and 85% pupae (dotted lines; Fig. 1a,b). Most of these WT and Ind pupae appeared within a 24-hour period after 3.6 and 4.9 days of larval development, respectively. In comparison, the two other juvenile diets (90° and øY) only produced 57% and 33% pupae, respectively. In the 90° and øY groups, larval development lasted 5.7 and 12.0 days and spanned over 12.1 and 17.3 days, respectively.

The pupa-to-adult developmental pattern diverged primarily between øY and the three other diets. To avoid bias resulting from earlier developmental variation, the proportion of emerging adults was estimated relative to the total number of pupae (plain lines; Fig. 1a). Whereas the great majority of WT, Ind and 90° adults emerged within a 2-3-day period, the emergence of øY adults spanned over two weeks. Although the numbers of 90° and øY adults were lower than those numbers of adults reared as larvae on the two other diets (Fig. 1c), the juvenile diet had little to no influence on pupa-to-adult development (see Fig. 1b,c and the correlation shown in Fig. S1). Significantly higher variability in the numbers of emerging pupae and adults was found for the øY-treated group than for the other three groups.

### Adult survival and weight

**Figure 2:**
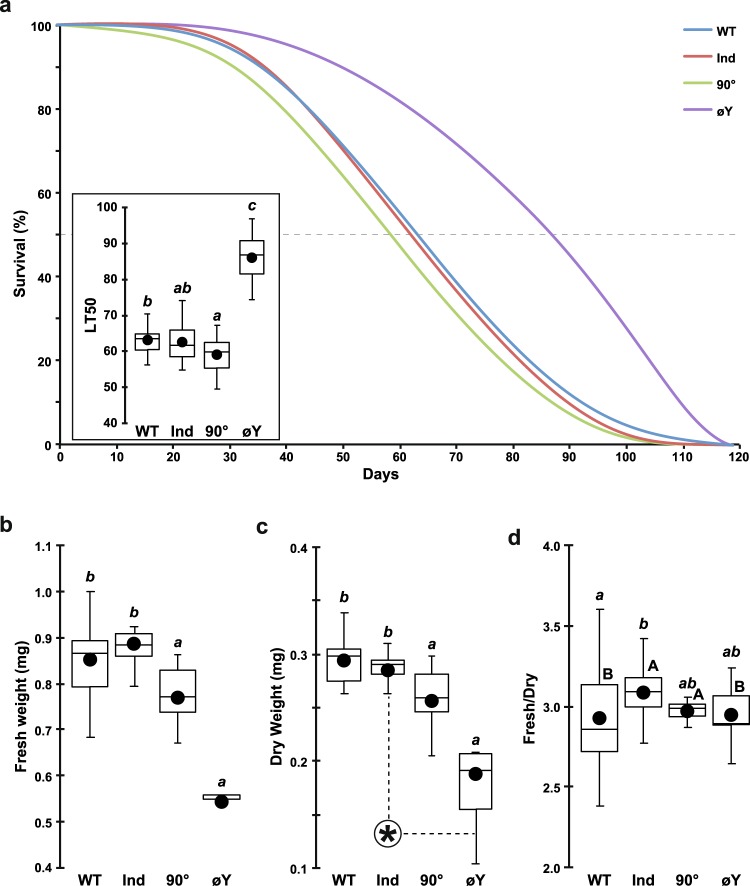
Adult survival and weight. (**a**) The survival curves show the frequency of surviving flies according to time (days) and juvenile diet. The inset shows the time (in days) at which 50% flies were dead (lethality time 50% = LT50). øY adults had an increased lifespan relative to adults reared on the three other juvenile diets. (**b**) We measured the weights of groups of freshly killed and (**c**) desiccated flies from the four juvenile diet groups. The data shown correspond to the estimated weight of individual flies (in g). (**d**) These data were used to estimate the fresh:dry weight ratio. (**a**) K(3 df) = 38.0, *p* < 10^−4^; Levene’s test F(3,70) = 1.8, *p* = 0.147; (**b**) K(3 df) = 39.9, *p* < 10^−4^; Levene’s test F(3,82) = 1.0, *p* = 0.392; (**c**) K(3 df) = 32.4, *p* < 10^−4^: Levene’s test F(3,79) = 3.75, *p* = 0.014; (**d**) K(3 df) = 9.8, *p* = 0.020; Levene’s test F(3,64) = 7.16, *p* = 0.0003). *N* = 20, 20, 20 and 14. For legends and statistics, please refer to Fig. 1 legend.

Next, we compared the survival of adult males reared on the four juvenile diets and maintained on Ind food. To estimate adult survival, we used the LT50 (lethality time for 50% individuals) and the slope (mortality rate/hour during the major lethality event). The survival curves of the WT, Ind and 90° flies were very similar (90° flies showed a slightly reduced LT50 relative to that of WT flies; see inset in Fig. 2a), while øY flies showed greatly extended survival (+30 days = 50% increase of LT50). These adults also showed a lower lethality rate compared to Ind individuals (slope; Fig. S2).

Given the variation observed both for pre-imaginal development (Fig. 1) and adult survival (Fig. 2a), we tested whether the variation could be related to fly ability to store nutriments and/or water. Therefore, we compared the weight of freshly killed and desiccated groups of flies. These values were also used to determine the fresh:dry weight ratio indicative of the amount of stored water. Despite the fact that 90° and øY flies showed significantly reduced fresh and dry weights compared to WT and Ind flies, their fresh:dry weight ratios did not differ from those of the other two groups (Fig. 2b–d). Our data also showed that Ind flies retained more water than did WT flies (Fig. 2d).

### Adult food preference

**Figure 3:**
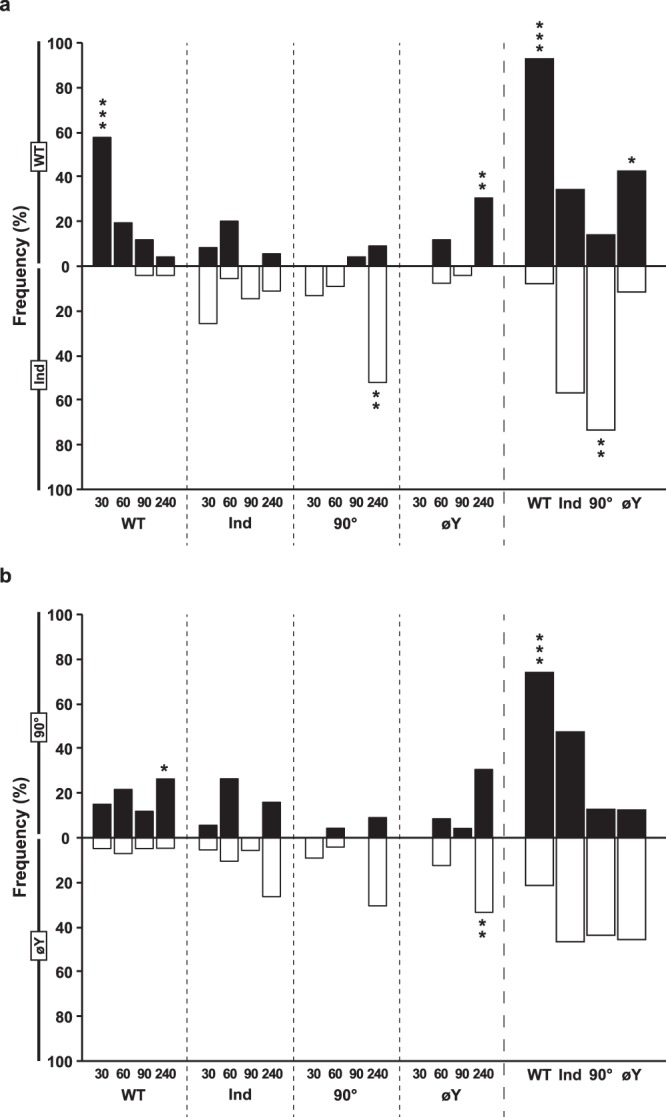
Adult food preference. Single starving male flies were introduced into a Y-maze olfactometer to test their preference to a dual food choice consisting of (**a**) “WT” *vs*. “Ind” diet or (**b**) “90°” *vs*. “øY” diet. The frequency of adults choosing either type of food was noted after 30, 60, 90 and 240 min of observation. Therefore, each “30” or “60” or “90” bar corresponds to a 30-min observation period, whereas the “240” bar corresponds to a 150-min period (between 90 and 240 min). The “240 cumulative” bars (right panels) present the pooled data from 0 to 240 min. The juvenile diets are indicated below each series of tests. Each pair of data was compared using Fisher’s exact test (***p < 0.001; **p < 0.01; *p < 0.05) *N* = 26, 32, 20, 14, 40, 18, 23 and 24.

Given the possible effects of juvenile diet on adult food preference^17^, we tested the responses of individual flies in a dual food choice experiment presenting either “WT/Ind” diets (Fig. 3a) or “90°/øY” diets (Fig. 3b). Food preference was evaluated during four time intervals (between 0–30, 30–60, 60–90 and 90–240 min), and the cumulative responses over the total test duration (240 min) were also determined. When presented with the “WT/Ind” choice, WT flies rapidly showed a preference for the WT diet (0–30 min; Fig. 3a), while Ind flies showed no preference, even after 240 min. 90° and øY flies showed slight preferences for the Ind and WT diet, respectively, during the 90–240 min period (such preference was also visible for the 240 min cumulative period).

When presented with the “90°/øY” food choice, WT flies showed a preference for the 90° diet during the 90–240 min period (which was also visible for the 240 cumulative period; Fig. 3b). In contrast, øY flies showed only a slight preference for øY food, observed during the 90–240 min period, but the effect was too weak to be detected for the cumulative period.

### Reproductive behaviour

Given that the microbiota and nutrient quality of the juvenile diet can affect reproduction^18,19^, we compared the mating ability, fertility, fecundity, and production of cuticular pheromones in male and female flies raised on the different juvenile diet treatments.

**Figure 4:**
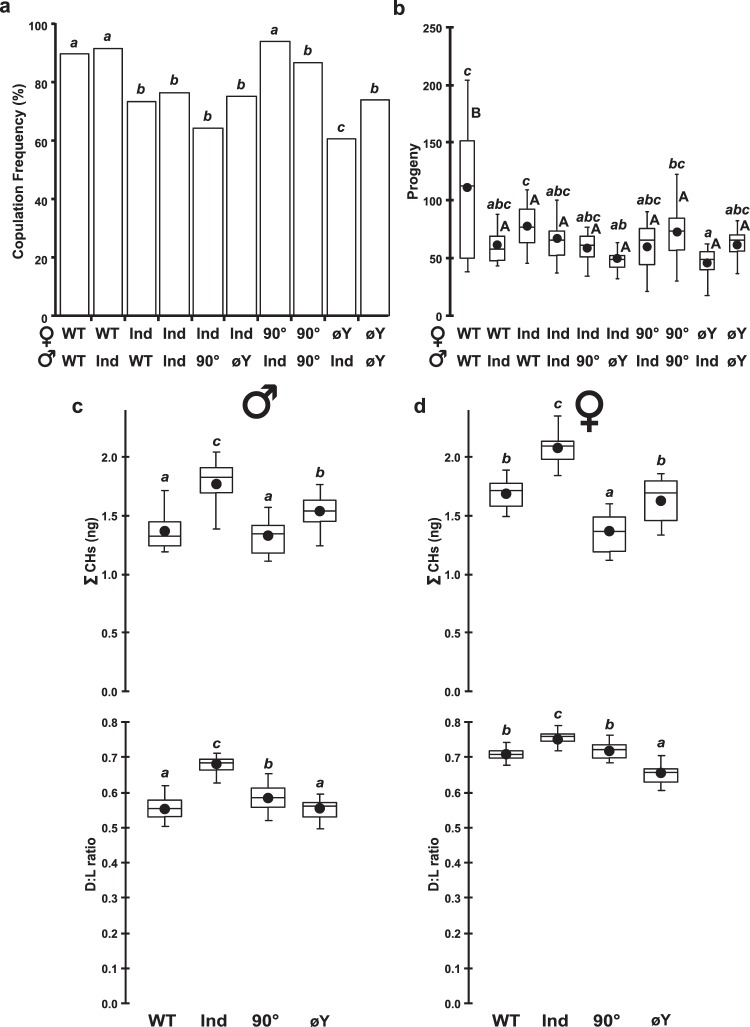
Reproduction-related characters. (**a**) The frequency of copulation during one hour and (**b**) fecundity were measured in male-female pairs (the juvenile diet of each fly is shown below each bar). The principal cuticular hydrocarbons (CHs) were measured in male (**c**) and female flies (**d**). Data shown correspond to the total absolute amount of CHs (∑CH in µg; top box-plots) and the ratio between Desaturated and Linear saturated CHs (D:L ratio). This ratio was calculated using the formula ([D − L]/[D + L]). Different letters indicate significant differences. Copulation frequencies (**a**) were compared using Wilks *G*^2^ likelihood ratio test and followed with a computation of significance by cell (G^2^(9df) = 17.1; *p* = 0.047), whereas means of other data were compared using the Kruskal Wallis test (**b**) K(9 df) = 38.8, *p* < 10^−4^; (**c**) K(9 df) = 38.8, *p* < 10^−4^ for ∑CHs and K(9 df) = 48.5, *p* < 10^−4^ for D:L ratio; (**d**) K(9 df) = 58.4, *p* < 10^−4^ for ∑CHs and K(9 df) = 51.9, *p* < 10^−4^ for D:L ratio), and their variances were compared using Levene’s test (**a**) F(3,76) = 1.2, *p* = 0.311 & F(3,76) = 1.6, *p* = 0.207; (**b**) F(3,76) = 2.7, *p* = 0.053 & F(3,76) = 0.7, *p* = 0.583). *N* = 20 for each diet and sex.

Copulation frequency was tested with pairs of 4-5-day-old flies over one hour. Given the high number of potential sex and treatment combinations, we only tested some of them (Fig. 4). Our data indicate that the juvenile diet influenced copulation frequency more strongly in females than in males. In particular, WT females (paired with either WT or Ind males) copulated more often (90–91%) than did Ind females (paired with all four types of males; 64–76%; Fig. 4a). A milder effect of juvenile diet was detected (*i*) in Ind males, which copulated more often than did 90° males with 90° females, and (*ii*) in øY males, which copulated more often than did Ind males with øY females. In addition, copulation duration significantly differed between pairs: IndxInd and IndxøY pairs showed longer copulation durations and greater variation of this parameter than did øYxøY pairs (Fig. S3A).

Fewer differences among pair combinations were noted for fecundity (number of adult progeny; Fig. 4b): this was likely due to the large variability of these data. The two pairs involving WT males (i.e., WT males with WT or Ind females) produced more adult progeny than did the pairs involving either Ind or øY males. Moreover, WTxWT pairs showed higher variability in fecundity than did the other pair combinations. No difference in fertility (ability to produce at least one progeny) or sex ratio (female:male progeny) was detected among the pair combinations (Fig. S3B,C).

We also compared female and male cuticular hydrocarbons (CHs), some of which have pheromonal roles, which might explain some of the mating differences described above (Fig. 4a). Both Ind males and females of Ind flies showed higher overall CH production (∑CHs; Fig. 4c,d) than that in same-sex flies of the other treatments. Moreover, the ratio between desaturated CHs and linear saturated CHs (Desat:Lin ratio) of male and female Ind flies was higher than that in same-sex flies of the other treatments (Fig. 4c,d). This effect was likely due to the increased production of desaturated CHs in Ind flies given that the linear CHs showed much less variation among treatments than did desaturated CHs except in øY flies, where they tended to be higher than in the three other treatments (Fig. S4).

## Discussion

Drosophila studies examining the influence of diet have generally focussed on the diet over all life stages (larva to adult) or only during adult life, and rarely have they focussed on the diet during larval development alone. Our study reveals that both yeast quantity and quality (varying among the different inactivation processes) in the juvenile diet influence *D*. *melanogaster* preimaginal development and several adult life traits.

Our developmental and aging data are supported by previous reports. Both the 90° and øY diets induced an increase in the duration of larva-to-pupa development and led to decreased pupal survival compared to the WT and Ind diets. While these developmental effects were stronger under the øY diet, the very few øY surviving adults showed an extended life span. Other studies have shown that in the absence of yeast, larval survival is extremely low (8% on grapes; 0% on a minimum medium^20^) but that a reduced amount of yeast in the larval diet can significantly increase adult life span^13^. Adult diet restriction can also increase life span^21,22^, which appears to be associated with the low amount of yeast^7^. Unlike the effect of larval diet restriction, the effect of adult diet restriction can be reversed when flies are returned to a yeast-rich medium^23^. Our results indicate that there is no direct relationship between preimaginal development and adult life span since 90° flies did not show an increased life span relative to Ind and WT flies. In the total absence of a sterol source, no Drosophila larvae can theoretically survive^24^. Therefore, the development of a few øY adults might have been due to the presence of sterols in the corn flour used in our basic food recipe. Moreover, the altered larva-to-pupa development induced by the 90° diet might be explained by the partial deterioration of sterols, possibly through oxidization caused by heating^12,25^. Indeed, the major sterol of yeast, ergosterol^26^, is sensitive to a 90° treatment^27^, whereas sterols remain unaltered in dehydrated inactive yeast such as that used in our “Ind” diet^28^. Alternatively, these differences could be caused by other non-sterol yeast components and/or to the transmission of yeast component(s) in the adult digestive tract varying between treatments. The persistence of live yeast from juvenile to adult stage could explain these differences (see Fig. S5). The latter hypothesis remains to be tested in adults resulting of individuals raised on alternative juvenile and adult diets switching between the different yeast foods tested here. Another approach would consist to raise individuals on a specific yeast diet only during a very restricted period of their development.

The nutritional requirements and preferences of Drosophila larvae and adults can differ, particularly in regard to sterols and fatty acids^29,30^. While *D*. *melanogaster* adults have long-term memory for food with nutritional value^31^, the specific nutrients encountered during pre-adult development can lead to adult preferences for the same nutrients, as shown for long chain fatty acids^17^. Bacteria colonizing the Drosophila digestive tract^32^ can influence the preferences of infested Drosophila for food that contains the same bacteria^33^. Moreover, yeast-bacteria interactions can change olfactory-driven behaviours in response to food containing associations of the two microbiota members^34^. Our food-preference assay indicates that the quality of the yeast in the larval diet influences olfactory-driven behaviour in Drosophila adults. In particular, a larval diet containing live yeast (WT) promotes a rapid and clear food preference for this diet in adult flies. This result has important implications for those laboratories that test olfactory responses in flies raised on Ind-type diet. Our data suggest that individuals developing in nature on live wild-type yeasts may show a different food preference response compared to flies raised in the laboratory.

Some of the bacteria present in the adult diet can influence Drosophila mating preferences for many generations. This phenomenon might be related to variation in cuticular hydrocarbons (CHs), some of which are known to have pheromonal effects^18,35^. While we found that the quality of yeast in the larval diet can affect copulatory behaviour and CH composition, we did not find a linear relationship between the two traits: WT females showed a higher copulation frequency than did Ind females (with males from similar treatments), while WT and 90° females paired with Ind males showed similar copulation rates. However, Ind flies of both sexes showed the highest CH amounts and the highest proportions of desaturated CHs (alkenes), compounds known to have pheromonal effects. Note that no relationship was found between the weight and the total CH amount (∑CHs). Interestingly, a recent study revealed a relationship between CH quality and lifespan^13^: Drosophila raised on a low-yeast diet showed a decreased production of autotoxins, represented by alkenes (desaturated CHs), which was correlated with an increased life span. Here, we found that øY females and, to a lesser extent, øY males produced lower alkene/alkane (D:L) ratio; these flies showed increased lifespan relative to the flies reared on the other three larval diets.

Additional in-depth studies are required to better understand the influences of different yeast treatments on the composition of the Drosophila diet and gut microbiota. The bacterial microbiota is known to affect many life traits of its host^36–38^, and the very early diet can change this microbiota over the long term^39^. Although most studies of microbiota have focused on bacteria, future studies should consider the interactions between bacteria and fungi, as recently shown^34^, to determine the roles of the dominant and persistent fungal species in these complex associations within the microbiota.

## Material and Methods

### Yeast strain and culture

We used the Saccharomyces cerevisiae strain BY4742 Wild Type (MATα his3Δ1 leu2Δ0 lys2Δ0 ura3Δ0) (EUROSCARF, Frankfurt, Germany). Preculture was performed over 48 h on Yeast extract (10 g/l)-Peptone (20 g/l)-Dextrose (20 g/l) medium (YPD) at 25 °C with rotation at 250 rpm with each yeast colony in a Erlenmeyer flask containing 100 ml of sterile YPD. A volume of the subculture was then transferred to an Erlenmeyer flask containing 100 ml of fresh YPD medium to reach 0.05 OD_600_. The culture was performed at 25 °C with 250 rpm rotation to allow growth to the early stationary phase.

### Food preparation

All juvenile food media contained corn flour (65.4 g/l) and agar (9.2 g/l), with S. cerevisiae yeasts mixed into the food for some media (adjusted to 5 × 10^8^ yeast cells/ml of food corresponding to 65 g/L yeast dry weight). The first medium contained live BY4742 S. cerevisiae wild type yeasts (“WT” = live wild type). The second medium contained another wild type strain of S. cerevisiae yeast produced by vacuum concentration and inactivated by spray drying (E50; Lesaffre Culinary Strasbourg, France; “Ind” = industrial treatment). The third medium contained BY4742 yeasts pasteurized at 90 °C for 15 min (“90°” = pasteurized treatment). The fourth medium received no yeast supplementation (“øY” = empty treatment). The different food types were consistently used within 48 h of production.

### Drosophila culture and egg collection

*D*. *melanogaster* strains were raised in 150-ml glass vials containing 50 ml of inactivated yeast/cornmeal/agar medium and maintained in a breeding room at 24.5 ± 0.5 °C with 65 ± 5% humidity on a 12:12 h light/dark cycle (subjective day from 8:00 am to 8:00 pm). Unless indicated, flies were transferred every two days to avoid larval competition and to regularly provide abundant progeny for testing. We used Dijon2000 (Di2), a wild-type strain maintained in our lab for 15 years, which showed very stable behavioural performance. Young emerging flies were housed in groups of 50–100 flies to allow mating in a vial containing 4 g of plain food. After 2–3 days, adults were transferred to a new vial for 3–4 h to allow egg laying. All of the experiments were performed at 25 °C.

### Preimaginal development

Fertilized eggs were transferred with a fine brush in groups of 50 to a vial containing one of each type of diet. In each vial, we measured the duration of development and scored the number of newly formed (white) pupae and of newly emerging male and female adults. All emerging adults were transferred to the regular lab medium (“Ind” treatment) for further testing. In most experiments, the adults were maintained in same-sex groups of 8–12 flies, but the males tested in copulation and food preference tests were isolated.

### Adult weight and survival

Adult survival was only determined for males since we did not control for the mating status of females and the presence of progeny affects female survival. Males held in small groups of 8–12 flies were transferred every 5–7 days to fresh food vials. The number of surviving males was scored at each transfer, and to maintain the group size as constant as possible, we pooled flies from vials with high mortality. Two measures of survivorship were recorded: LT50 (time at which 50% of the flies had died) and lethality slope (the steepness of this curve indicates the proportion of flies dying per hour).

We weighed groups of 10 live anaesthetized females on a precision balance (±10 µg; Sartorius R160-P) to obtain their fresh weight. Each group of females was then placed for 24 h in an empty glass vial in a 37 °C dry incubator to allow complete desiccation. The dead, dry flies were then weighed to obtain the dry weight. The relative level of water in each group was estimated based on the fresh weight:dry weight ratio.

### Reproduction

We measured the copulatory ability of pairs of 4-day old flies. One male was aspirated (without anaesthesia) under a watch glass used as an observation chamber (1.6 cm^3^). After 10 min, a virgin female was introduced. Each test was performed, between 9 am and noon, for 60 min under white light, and the overall frequency of copulation events was measured for each pair. We calculated the cumulative proportion of flies mating during the observation period and the duration of copulation events (time between copulation onset and separation). Females mating within the one-hour observation period were transferred alone to a fresh food vial (the male was immediately discarded), while non-mating pairs were individually maintained in a food vial (with the male discarded 24 h later). In all cases, the presence (fertility), number (fecundity) and sex ratio of adult progeny were noted.

### Food preference

For food preference assay, we used 5-day old males that had been individually maintained in the preceding 24 h in empty vial containing only a humid filter paper. After a brief anaesthesia on ice, individual males were individually introduced into the straight arm of a Y-shape olfactometer. Tests were always performed between 9 am and 1 pm. At the end of the straight arm, the device is divided in two additional arms, each one containing a filter paper previously incubated at 25 °C for 30 min with one of two types of food medium (WT *vs*. Ind or 90° *vs*. øY). The test was performed over a 240-min period under far-red light (LED bulbs). After the test start, every 30 min, the position of the fly in the device was noted relative to the arm (type of food) chosen. Flies were rarely seen retreating into the straight arm of the device. Flies from different yeast treatments were simultaneously tested. Between trials, the substance choices were alternated between the olfactometer arms and assays. All of the experiments were performed between 8 am and noon. Significant differences were determined with the Fisher exact test.

### Cuticular hydrocarbons

Five-day-old flies were frozen for 5 min at −20 °C and individually extracted for 5 min at room temperature using 30 µl of a mixture of hexane and methylene chloride (50/50; vol/vol) to extract a large variety of compounds. The solution also contained 3.33 ng/µl of C26 (*n*-hexacosane) and 3.33 ng/µl of C30 (*n*-triacontane) as internal standards. Cuticular hydrocarbons were quantified by gas chromatography using a Varian CP3380 gas chromatograph fitted with a flame ionization detector, a CP Sil 5CB column (25 m by 0.25-mm (internal diameter); 0.1 µm film thickness; Agilent) and a split–splitless injector (60 ml/min split-flow; valve opening 30 sec after injection) with helium as the carrier gas (50 cm/sec at 120 °C). The temperature program began at 120 °C, ramping at 10 °C/min to 140 °C, then ramping at 2 °C/min to 290 °C and holding for 10 min. Individual CH profiles were determined by integration of 46 peak areas in males and females, representing all of the peaks that could consistently be identified in all individuals. The chemical identity of the peaks was determined using gas chromatography–mass spectrometry system equipped with a CP Sil 5CB column. The amount (ng/insect) of each component was calculated based on the readings obtained from the internal standards. Twenty flies were tested per condition.

### Statistics

All statistical analyses were performed using XLSTAT 2012^40^. For each diet, logistic regression was used to characterize the relationship between mortality and time by estimating the LT50 and regression slope^41^. Thereafter, an inter-diet comparison for these two parameters was carried out using a Kruskal-Wallis test with Conover-Iman multiple pairwise comparisons (*p* = 0.05, with Bonferroni correction) after excluding extreme outliers using Tukey’s method^42^.

The number of pupae and adults, the overall amount of CH (∑CH), the Desaturated:Linear ratio (D:L), the fresh and dry weights and their ratio, the total number of adult progeny and the sex ratio were also compared using the same statistical tests. Variances were compared using Levene’s test involving absolute deviations at the median followed by post-hoc multiple pairwise comparisons (*p* = 0.05, with Bonferroni correction). Freque ncies were compared using either a Fisher’s exact test (pair comparison) or a Wilks *G*^2^ likelihood ratio test completed with a computation of significance by cell (Fisher’s exact test).

## Electronic supplementary material

Supplementary information

## Data Availability

The datasets generated during and/or analyzed during the current study are available at the following address https://figshare.com/s/f31063b74e4049b5d07b.
