# Supplementary material for: Yeast quality in juvenile diet affects Drosophila melanogaster adult life traits
Source: Sci Rep. 2018 Aug 30;8:13070. doi: 10.1038/s41598-018-31561-9 (PMC6117321; doi:10.1038/s41598-018-31561-9)
Supplement: Supplementary file 1 — Supplementary information [file 41598_2018_31561_MOESM1_ESM.pdf]

Yeast quality in juvenile diet affects

Drosophila melanogaster adult life traits

Cédric Grangeteau[1], Fairouz Yahou[1,2], Claude Everaerts[2], Sébastien Dupont[1], Jean-Pierre Farine[2], Laurent Beney[1]*, Jean-François Ferveur[2]*

SUPPLEMENTAL MATERIAL AND METHODS

Yeast strain and culture. We used the Saccharomyces cerevisiae strain BY4742 transformed[1] with the YIp211-SUR7GFP plasmid[2]. Culture conditions were identical to conditions used for WT strain.

Food preparation. Food medium with GFP-tagged yeasts was prepared similarly to that described for BY4742 WT strain. This food medium contained corn flour (65.4 g/l) and agar (9.2 g/l), with GFP-tagged *S. cerevisiae* yeasts mixed into the food medium (adjusted to 5×108 yeast cells/ml of food corresponding to 65 g/L yeast dry weight).

Drosophila culture. Fertilized eggs were transferred with a fine brush in groups of 50 to a vial containing diet prepared with GFP-tagged yeasts. L2 stage larvae were collected and rinsed 3 times with distilled water. Larvae were then transferred on inactivated yeast/cornmeal/agar medium and maintained in a breeding room at 24.5±0.5°C with 65±5% humidity on a 12:12 h light/dark cycle (subjective day from 8:00 am to 8:00 pm) until adult eclosion.

Drosophila dissection and two-photon microscopy observation. Drosophila digestive tract was dissected out of Drosophila flies (3-days old) using a pair of fine dissection tweezers (FST- #11253-20, Dumont Switzerland). Observations were carried out on the whole digestive tract placed on a glass plate with PBS and covered with a fine glass slide. Anatomic observation was performed under a Nikon A1-MP scanning two-photon microscope (Nikon, Japan) with a x60 Apo infrared (IR) objective (NA: 1.27, Water Immersion, Nikon, Japan). Optical band-pass filters (FF01-492/SP-25, FF03-525/50-25, Semrock) with 25 nm bandwidths and center at 445 was used to collect the fluorescence. Excitation was provided by an Coherent Chameleon Vision II mode-locked femtosecond Ti:sapphire laser (140 fs pulse duration, 80 mHz) at 830 nm.

LEGENDS FOR SUPPLEMENTARY FIGURES

Figure S1. Correlation between the number of pupae (∑P) and number of adults (∑Ad). Each data point corresponds to the values observed in each vial. The data from all juvenile diet treatments were pooled (see Fig. 1).

Figure S2. Slopes of adult survival curves. Each value indicates the proportion of flies dying per hour for each juvenile diet (see Fig. 2A).

Figure S3. Reproduction parameters of pairs subjected to copulation tests. (**A**) We determined the copulation duration of pairs tested (see Fig. 4A). Females used in these tests were individually kept in food vials. (**B**) Fertility was scored if at least one adult progeny was left in the vial. (**C**) The sex ratio corresponds to the ratio of female to male flies (F/M) emerging in each vial. No significant difference was found for either parameter (see Fig. 4A&B). For statistics, please refer to Fig. 4.

Figure S4. Production of cuticular hydrocarbons. Principal cuticular hydrocarbons (CHs) were measured in male and female flies. We calculated the total absolute amount of Desaturated CHs (∑Desat; top plots) and Linear saturated CHs (∑Lin; bottom plots) in ng. For statistics, please refer to Fig. 4.

Figure S5. Visualisation of GFP-tagged yeast in the digestive tract of Drosophila flies fed with GFP-tagged live yeast during juvenile stage. Cells of intestinal tract are visible by green auto-fluorescence. Yeast cells are visible in green thanks to tag with GFP of Sur7 proteins. A large number of intact yeasts is visible in the crop and, to a lower extent, in other parts of the gut. The white bar scale represents 200 µm.

REFERENCES

1. Dupont, S., Beney, L., Ritt, J. F., Lherminier, J. & Gervais, P. Lateral reorganization of plasma membrane is involved in the yeast resistance to severe dehydration. *Biochim. Biophys. Acta - Biomembr.* **1798,** 975–985 (2010).
2. Grossmann, G., Opekarová, M., Malinsky, J., Weig-Meckl, I. & Tanner, W. Membrane potential governs lateral segregation of plasma membrane proteins and lipids in yeast. *EMBO J.* **26,** 1–8 (2007).

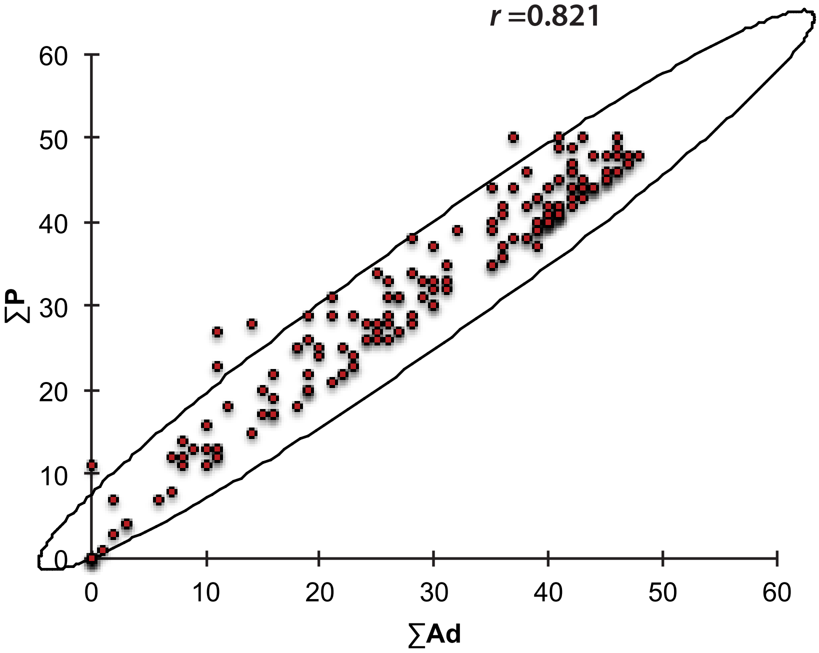

r =0.821
60
50
40
30
20
10
0
∑P
0
10
20
30
40
50
60
∑Ad

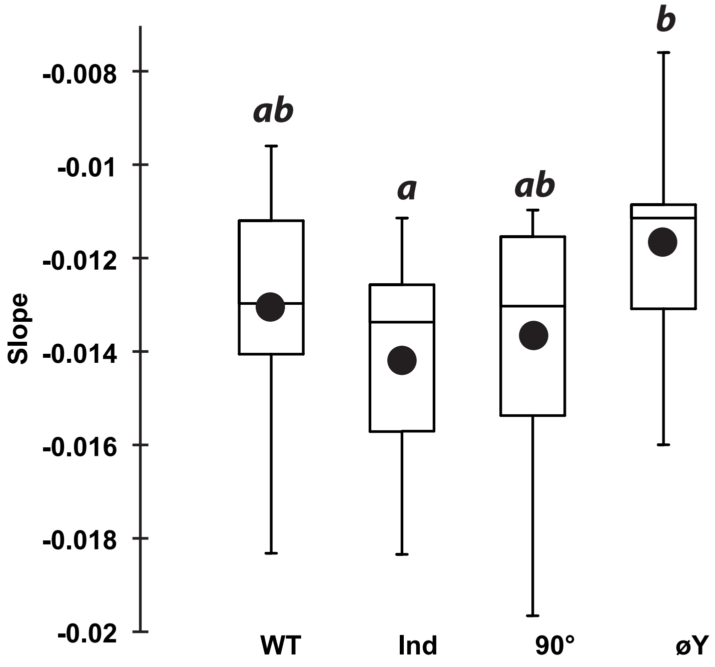

b
ab
a
ab
-0.008
-0.01
-0.012
-0.014
-0.016
-0.018
-0.02
Slope
WT
Ind
90°
øY

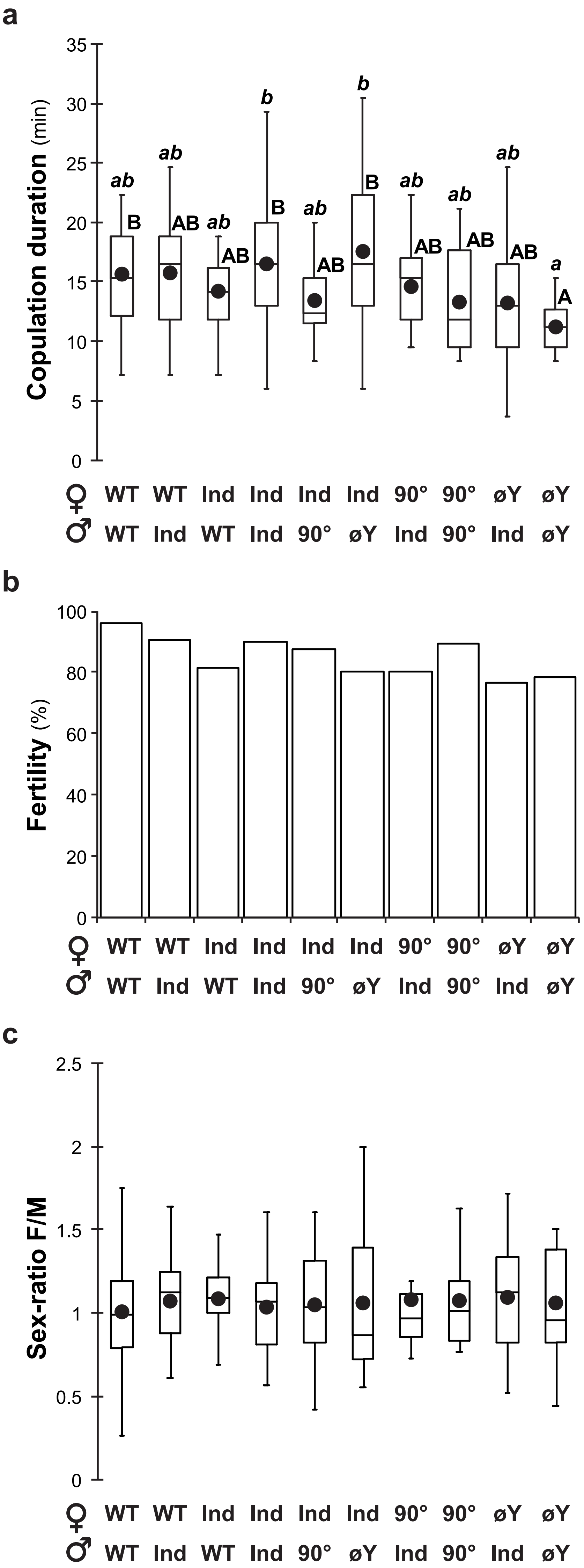
a
Copulation duration (min)
35
30
25
20
15
10
5
0
ab
B
AB
ab
AB
b
B
ab
AB
b
B
ab
AB
ab
AB
ab
AB
a
A
♀ WT WT Ind Ind Ind Ind 90° 90° øY øY
♂ WT Ind WT Ind 90° øY Ind 90° Ind øY
b
Fertility (%)
100
80
60
40
20
0
♀ WT WT Ind Ind Ind Ind 90° 90° øY øY
♂ WT Ind WT Ind 90° øY Ind 90° Ind øY
c
Sex-ratio F/M
2.5
2
1.5
1
0.5
0
♀ WT WT Ind Ind Ind Ind 90° 90° øY øY
♂ WT Ind WT Ind 90° øY Ind 90° Ind øY

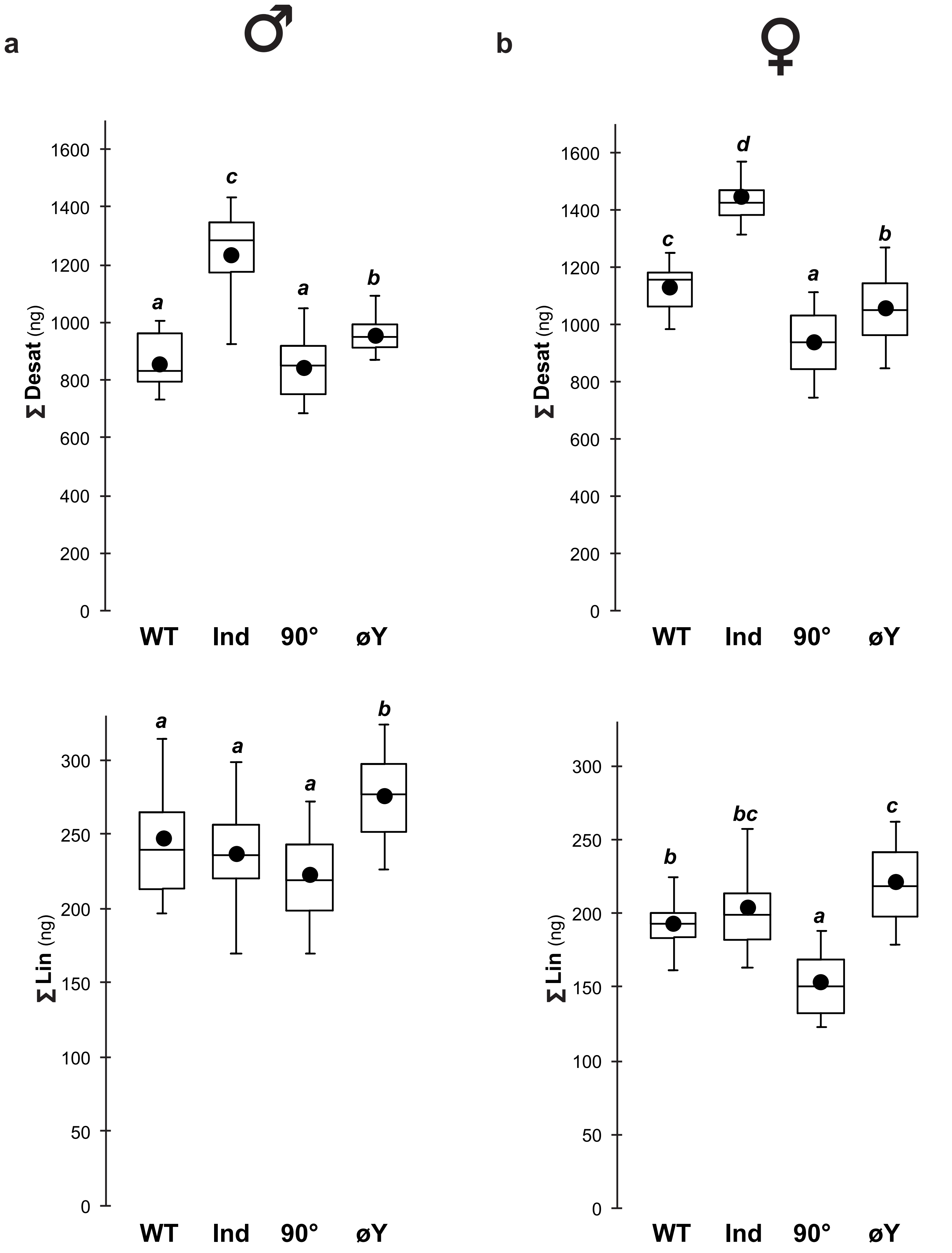

a
♂
b
♀
Σ Desat (ng)
0
200
400
600
800
1000
1200
1400
1600
a
c
a
b
c
d
a
b
WT
Ind
90°
øY
Σ Lin (ng)
0
50
100
150
200
250
300
a
a
a
b
b
bc
a
c

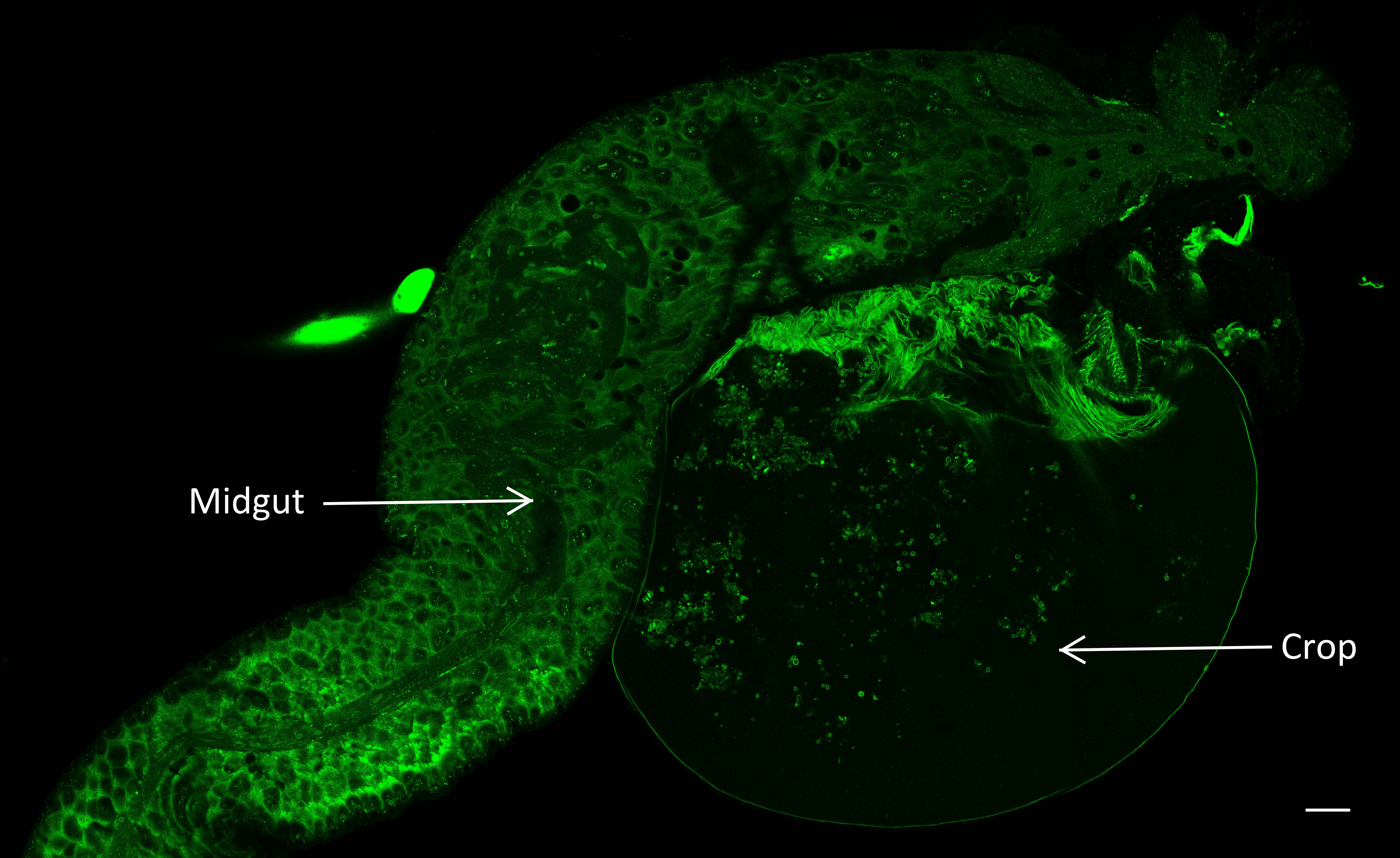
Midgut
Crop
